# Ultrasound-Guided Percutaneous Arteriovenous Fistula Creation Simulation Training in a Lifelike Flow Model

**DOI:** 10.3390/bioengineering9110659

**Published:** 2022-11-06

**Authors:** Andrej Isaak, Thomas Wolff, Andrei Zdoroveac, Fadi Taher, Lorenz Gürke, Sabine Richarz, Shuaib Akifi

**Affiliations:** 1 Department of Vascular and Endovascular Surgery, University Hospital Basel, Spitalstrasse 21, 4031 Basel, Switzerland; 2 Vascular and Endovascular Surgery, Cantonal Hospital Aarau, 5001 Aarau, Switzerland; 3 Vascular and Endovascular Surgery, Klinik Ottakring, Montlearstrasse 37, 1160 Wien, Austria

**Keywords:** simulation training, ultrasonography, interventional, arteriovenous fistula, percutaneous

## Abstract

*Objectives:* To assess the feasibility and training effect of simulation training for ultrasound-guided percutaneous arteriovenous fistula (pAVF) creation in a lifelike flow model. *Methods:* Twenty vascular trainees and specialists were shown an instructional video on creating a pAVF in a dedicated flow model and then randomized to a study or control group. The procedure was divided into five clearly defined steps. Two observers rated the performance on each step, and the time to perform the exercise was recorded. The study group participants underwent supervised hands-on training on the model before performing a second rated pAVF creation. All participants subsequently completed a feedback questionnaire. *Results:* After supervised simulation training, the study groups participants increased their mean performance rating from 2.2 ± 0.9 to 3.2 ± 0.7. A mean of 3.8 ± 0.8 procedure steps was accomplished independently (control group 2.1 ± 1.4; *p* < 0.05). The time taken to perform the procedure was 15.6 ± 3.8 min in the study group (control group 27.2 ± 7.3, *p* < 0.05). The participants with previous experience in ultrasound-guided vascular procedures (n = 5) achieved higher overall mean scores 3.0 ± 0.8 and accomplished more steps without assistance (2.0 ± 1.0) during the simulation training compared to their inexperienced peers (1.5 ± 0.3 and 0.8 ± 0.4, respectively). The feedback questionnaire revealed that the study group participants strongly agreed (n = 7) or agreed (n = 3) that training on the simulation model improved their skills regarding catheter handling. *Conclusions:* The study group participants increased their overall performance after training on the simulator. More experienced attendees performed better from the beginning, indicating the model to be lifelike and a potential skill assessment tool. Simulation training for pAVF creation using a lifelike model may be an intermediate step between acquiring ultrasound and theoretical pAVF skills and procedure guidance in theatre. However, this type of training is limited by its reliance on the simulator quality, demonstration devices and costs.

## 1. Introduction

The ultrasound-guided creation of a percutaneous arteriovenous fistula (pAVF) between the deep communicating vein (DCV) in the proximal forearm and the proximal radial artery (PRA) for hemodialysis access is a relatively novel procedure with promising technical success and patency rates [1,2,3]. However, vascular surgeons trained in convention vascular access surgery may find pAVF creation challenging, particularly because it requires skills in vascular ultrasound (VUS) for the preoperative evaluation and for the ultrasound-guided maneuvers during the procedure. It has previously been shown how simulation training may improve the acquisition of skills for ultrasound-guided vessel punctures [4]. For instance, training on a vessel phantom effectively improved the first attempt success rate for ultrasound-guided radial artery cannulation in real patients. A repeated training curriculum accelerated the learning curve for recall skill proficiency and reduced the interindividual variability of skill acquisition. However, to date, no dedicated training tool to simulate VUS-guided pAVF creation in the proximal forearm with the ability to simulate flow and adequate visualization has been available. The current study aims to describe and validate a simulation training of a VUS-guided pAVF creation in a lifelike flow model developed specifically for this purpose.

## 2. Materials and Methods

### 2.1. Flow Model Development

**Figure 1:**
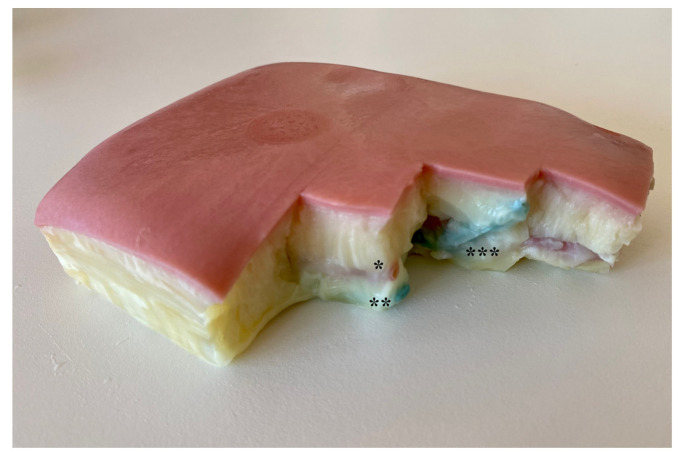
pAVF simulation model inlay. Legend: hydrogel inlay with embedded silicone artery *, vein ** and vessel crossing ***.

**Figure 2:**
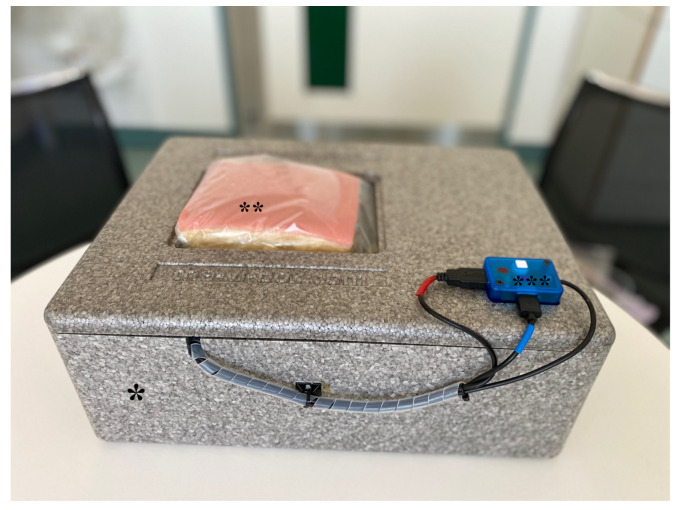
Complete pAVF simulation model. Legend: simulation model box * with hydrogel inlay ** and pump ***.

The model was developed in collaboration with Vascular International School https://vascular-international.org/ (accessed on 26 September 2022). The surface of the model resembling human skin is designed to represent the cubital fossa and region of the proximal forearm (LifeLike Biotissue, London, ON, Canada, https://lifelikebiotissue.com/ (accessed on 26 September 2022)). Embedded in the model are polyvinyl tubes simulating the cephalic vein (CV), the deep communicating vein (DCV) and the proximal radial artery (PRA). The crossing point between the DCV and PRA lies at a depth of approx. 3 cm, and the distance between the DCV and PRA is approx. 1–2 mm (Figure 1). A total of three “vessel” pairs are embedded in the hydrogel inlay. The inner vessel diameters of the PRA and DCV are approx. 3–4 and 4–5 mm, respectively. Each model contains three sets of vessels so the exercise can be performed three times on each model. The model is placed in a box with a custom-made styrodur frame and connected to a unidirectional pump system (ANSELF, Micro DC brushless water circulating pump, DC5V USB, Model DC30B, Taizhou, China), delivering pulsatile flow with water through the “arterial” tube and continuous flow through the venous “tube” (Figure 2). The entire model contains no air and has similar characteristics as human soft tissue, rendering the visualization of the imbedded structures by VUS, the VUS-guided puncture of the vessels and the VUS-guided handling of the endovascular appliances very lifelike. The development of a similar predecessor model has been presented in previous publications and was validated for closure device simulation training [5].

### 2.2. Study Design and Intervention

**Figure 3:**
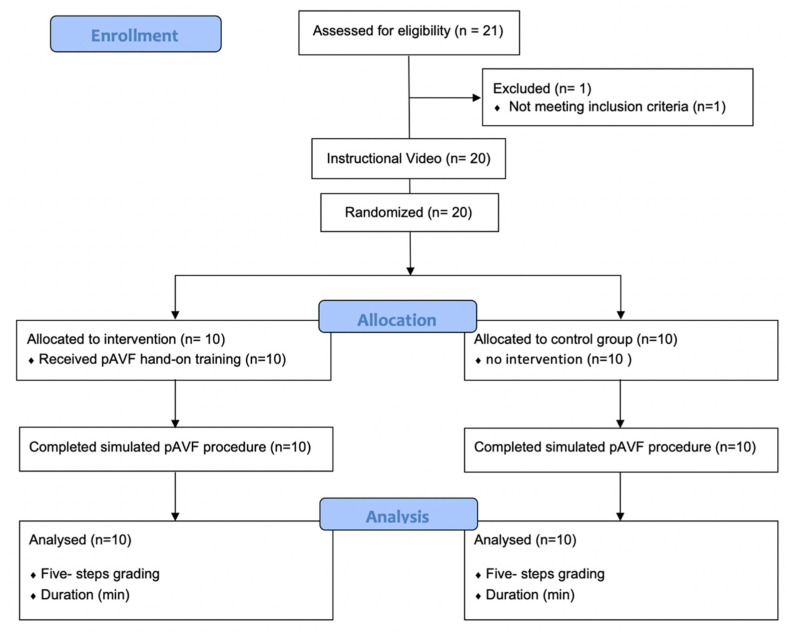
Consort flow diagram.

**Figure 4:**
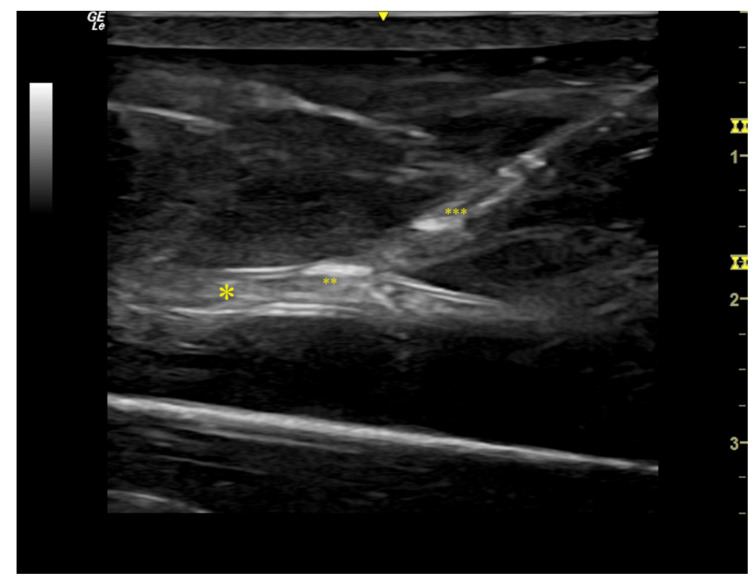
Vascular ultrasound imaging of pAVF training. Legend: positioning of Ellipsys^®^ catheter at anastomosis site, simulated artery *, arterial ** and venous *** device part, ▼ transducer`s center marker.

Residents and qualified vascular interventionalists and surgeons, inexperienced in pAVF creation, were randomized into a study and control group (Figure 3). Both groups received theoretical instructions by a tutor on how to perform VUS-guided pAVF creation and watched a video of a pAVF procedure performed on our model. The participants of the study group additionally received step-by-step, hands-on training on the pAVF model assisted by an expert without time limits and where each candidate performed an entire pAVF procedure, including all steps, at least once. Participants of both groups then performed a pAVF procedure on the model under surveillance. The procedure was divided into five steps: 1. visualization of DCV and PRA in longitudinal and cross-section view (general VUS skills); 2. VUS-guided puncture and VUS-guided crossing from the DPV into the PRA (Cook Percutaneous Entry Thinwall Needle- 21 G/7 cm, Limerick, Munster, Ireland); 3. VUS-guided guide-wire advancement and sheath insertion (Terumo Glidesheath Slender™ 6F, Terumo Deutschland GmbH, Eschborn, Zweigniederlassung Spreitenbach, Switzerland); 4. handling of the vascular access catheter (Medtronic, Münchenbuchsee, Switzerland, Ellipsys™ Vascular Access System, Figure 4); and 5. VUS-guided anastomosis dilatation with a 5 × 20 mm^2^ PTA balloon (Boston Scientific, Ecublens, Switzerland, 40 cm Shaft Sterling Balloon). The performance of each step was graded by two experts (AI and MR) on a scale from 1–5, with 1 representing the skills of a beginner and 5 those of an expert. This was undertaken to determine content validity and the appropriateness of the model realistically to teach what it is supposed to teach. It was documented whether the candidates were able to perform the tasks independently or whether they needed assistance.

An overall score was calculated by taking the mean of the individual scores for each task. Participants were video recorded, and the time from starting sonography to removing all devices was recorded. The VUS device (GE Medical Systems, LOGIQ e, SN 474659WX0, Wuxi, China) was preadjusted to ideal settings for every participant. To avoid any bias, no feedback was given to the candidates.

Face validity in terms of the realism of the simulator was assessed by evaluating a feedback questionnaire completed by all participants after the training.

### 2.3. Ethical Considerations

The study adhered to the principles of the World Medical Association included in the Declaration of Helsinki [6]. The highest level of professional behavior and confidentiality was applied, and the relevant national laws for data protection were fulfilled. All participants were volunteers, signed an informed consent and completed a questionnaire evaluating the flow model and simulation training. No ethical approval was necessary for this type of simulation training study, which did not include any patients.

### 2.4. Statistical Analysis

*T*-test and paired *t*-test were used to compare means in continuous variables, with nonparametric equivalents (Mann-Whitney U and Wilcoxon tests) used when applicable. Categorical values were compared between groups using Fisher’s exact test. Statistical analysis was performed using IBM SPSS Statistics (IBM Statistics for Windows, Version 24.0. Armonk, NY, USA).

According to the results of a previous study [5], a sample size of 20 participants with an expected mean difference of 16% regarding overall performance between groups (two-tailed significance level of 0.05, 80% power) was calculated.

## 3. Results

A total of 20 participants were randomized (Randomizer for Clinical Trial App, block size 2) into two groups (n = 10 per group). One eligible trainee who declared to have performed a pAVF procedure prior to simulation training was not randomized. The female attendees accounted for 35% (n = 7). The mean age of the study group was 43 years (±13.8), with no significant difference from the control group (40 ± 13.2, *p* = 0.57).

**Figure 5:**
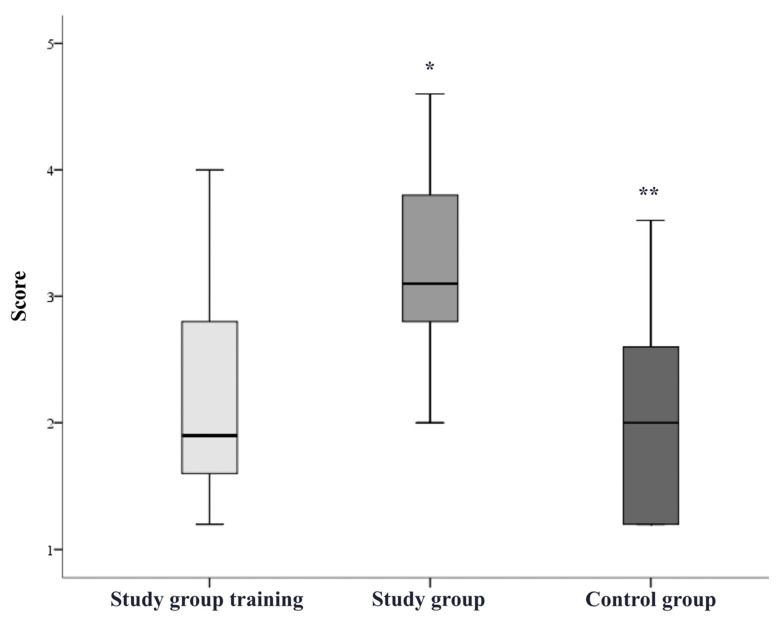
Overall grading results. Legend: score range 1–5, * *p* < 0.05 compared to study group training, ** *p* < 0.05 compared to study group.

**Figure 6:**
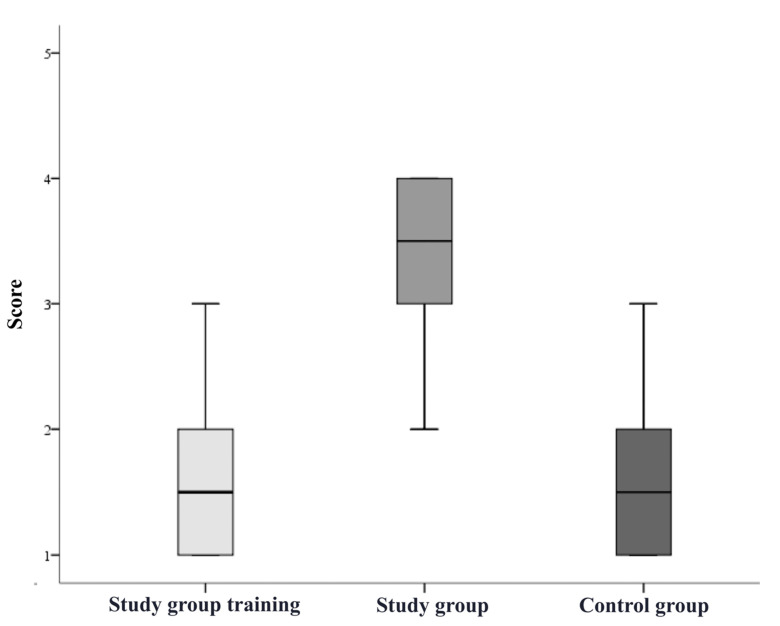
Grading of catheter handling. Legend: score range 1–5.

**Figure 7:**
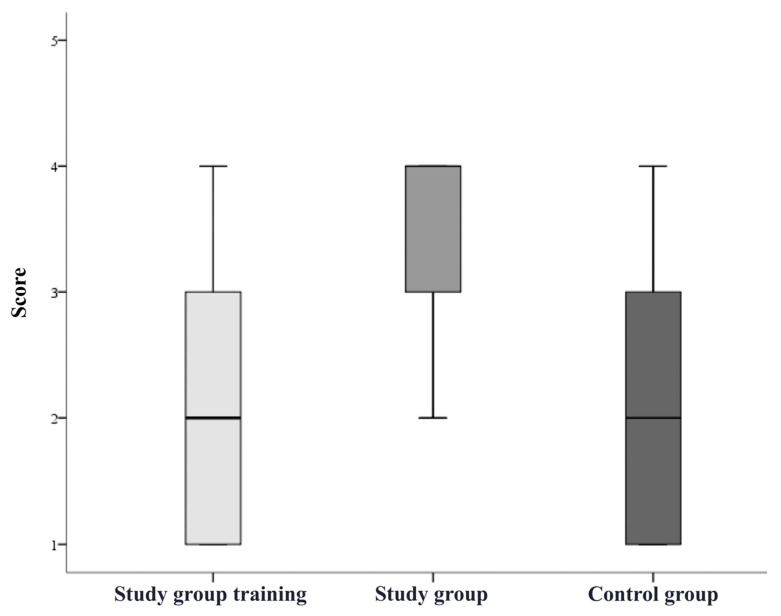
Grading of VUS-guided angioplasty of anastomosis. Legend: score range 1–5.

**Figure 8:**
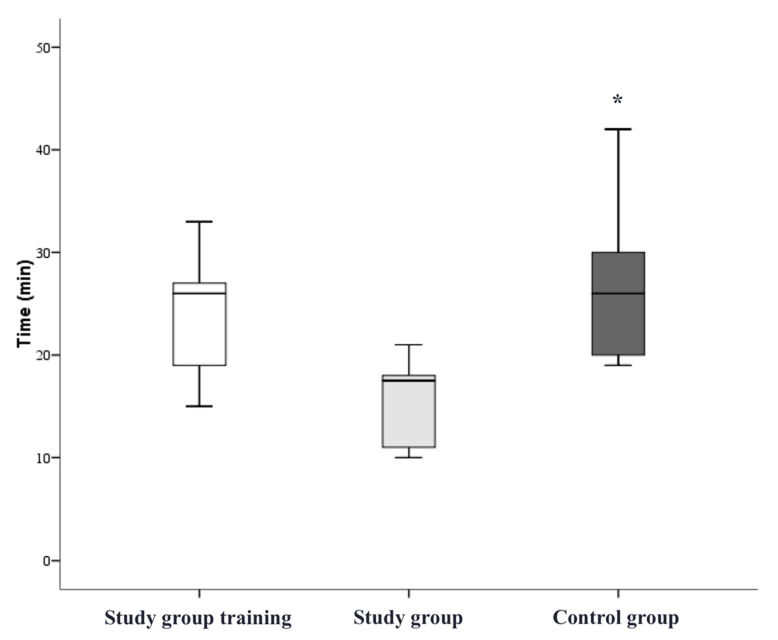
Mean duration of training and simulated pAVF creation. Legend: * *p* < 0.05 compared to study group.

The participants in the study group significantly improved their overall mean rating after completing the simulation training (overall score before training 2.2 ± 0.9, after training 3.2 ± 0.7; *p* < 0.05, Figure 5). The grading results of each step during the training of the study group and during the pAVF simulation by both groups were recorded and showed a notable learning effect in catheter handling and the VUS-guided angioplasty of anastomosis (Figure 6 and Figure 7). The study group participants significantly increased the number of tasks successfully performed without assistance (during training 1.4 ± 1.0, after training 3.8 ± 0.8; *p* < 0.05). The most marked improvements were seen for performance in VUS-guided puncture, vascular access catheter handling and pAVF dilatation (Table 1). The average time required for the pAVF procedure decreased from 24.6 ± 5.6 to 15.6 ± 3.8 min after training (Figure 8).

The control group participants, without simulation training, were able to perform a mean of 2.1 ± 1.4 tasks independently (vs. 3.8 ± 0.8 study participants after simulation training) with an overall mean score of 2.0 ± 0.8 (*p* < 0.05 vs. 3.2 ± 0.7 study participants after simulation training, Figure 5). The time required for the pAVF creation was 27.2 ± 7.3 min in the control, which was significantly longer than in the study group after training (15.6 ± 3.8, *p* < 0.05, Figure 8).

Eight participants (40%), five in the study and three in the control group (*p* = 0.65), had previous experience in VUS-guided vascular procedures, performing more than 40 of such interventions per year. The experienced participants in the study group achieved higher overall scores than their unexperienced peers on the overall five-point performance scale during simulation training (overall mean scores 3.0 ± 0.8 versus 1.5 ± 0.3, respectively; *p* < 0.05) and successfully accomplished 2.0 ± 1.0 out of five tasks (unexperienced participants, 0.8 ± 0.4; *p* < 0.05).

**Figure 9:**
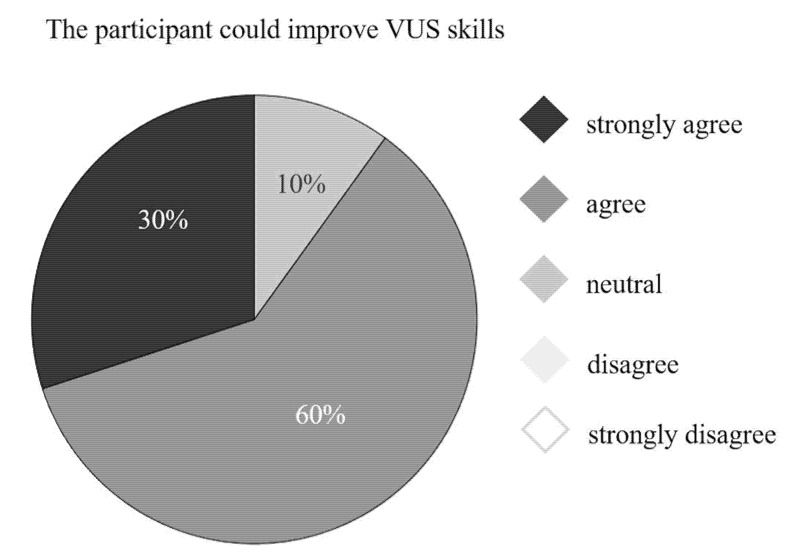
Improvement in VUS skills according to feedback questionnaire.

The evaluation of the feedback questionnaire revealed that participants strongly agreed (n = 11) or agreed (n = 9) that the simulation flow model was lifelike. Sixty percent of participants stated their VUS skills improved (Figure 9). All study group participants strongly agreed (n = 7) or agreed (n = 3) that they had improved their skills regarding vascular access catheter handling, and all would highly recommend pAVF simulation training using the lifelike flow model. 

## 4. Discussion

To our knowledge, this is the first study to examine the feasibility and impact of simulation training for VUS-guided pAVF creation in a lifelike model. Our findings show that the developed flow model of the cubital fossa presented lifelike characteristics to simulate and aid the training of pAVF creation. Trainees and experienced vascular surgeons were able to perform an entire pAVF procedure step by step to develop their procedural skills. Simulation training led to a significant improvement in performance, a reduction in the assistance required and a reduction in the procedure duration. In particular, training improved the performance of the demanding VUS-guided steps of the procedure, such as VUS-guided crossing from the DCV to the PRA and the handling of the vascular access catheter, emphasizing the content validity and the appropriateness of the simulator as a teaching modality. For the independent raters, it was astounding to observe how the confidence of the participants increased during the training. In addition, a performance difference between the experienced and inexperienced participants could already be determined during the first pAVF simulation attempt. The participants gave very positive feedback regarding the lifelike properties of the model, including a realistic haptic feedback and excellent VUS visualization to guide all maneuvers, demonstrating face validity.

pAVF with Ellipsys™ vascular access system has become an additional option for hemodialysis vascular access creation. All advocates of pAVF insist that extensive experience in VUS-guided vessel puncture and general excellent VUS skills to evaluate the vessel anatomy in the elbow for suitability for pAVF [7] are key to mastering the new technique. Accordingly, the proctoring of the first pAVF cases by experts has been described as beneficial [8]. While VUS skills to evaluate vascular anatomy and the suitability for pAVF creation can be easily exercised on patients, it is more difficult to practice the pAVF creation step by step on patients. Here simulation training in a lifelike model offers an important advantage.

Numerous models and training programs have been developed to train VUS-guided procedures [9,10,11]. In particular, pediatric VUS-guided vascular cannulation models were introduced in order to address the challenges of small vessels and the high complication rates in this patient population. The feasibility and effectiveness of training for simple steps such as vascular cannulation [12] using chicken breast and silicone tubes to achieve a lifelike setting have been shown [13]. We adopted flow models for open surgical procedures [5] to facilitate complex ultrasound-guided maneuvers to avoid cadaveric tissue and guarantee reproducibility. The main challenges when developing the pAVF model were ensuring that the vessels had a maximum distance of 2 mm at the level of the crossing between the vein and artery and finding materials for the vessels and the surrounding tissue that would closely mimic human tissue and performed reproducibly. Other important aspects of our model are the detectable flow in the vessels and the realistic haptic feedback during puncture and device handling. For instance, we observed how a failed puncture during training led to vessel perforation and subsequent extravasal fluid collection, which complicated the ensuing simulated pAVF procedure. However, simulation and reproduction of a vessel spasm on the model after puncture failure was not achieved.

Augmented reality (AR) sonographic devices have been shown to decrease the time of a successful cannulation of a vascular structure on a phantom during vascular access training [14,15,16]. Incorporating AR technology to achieve a 3D view of the model might further improve training on our model as well as facilitate a pAVF creation in real life.

So far, achieving competency in VUS is not compulsory for vascular surgery residents in training [17]. Therefore, it was not surprising that the majority of the participants failed to successfully create a simulated pAVF on their first attempt. As open surgical and endovascular procedures in combination with different intraoperative imaging modalities become increasingly complex, standardized step-by-step simulations on lifelike models have become an increasingly important part of surgical training [18]. During the last decade, several simulation models have been developed, including a hybrid procedure suite, a hybrid ex vivo placenta-human skull simulator for cerebral bypass training, a live tissue simulator hybrid porcine model for REBOA placement and lifelike pulsatile flow models [19,20,21,22]. All settings have the aim to increase the confidence of the surgeon in a safe environment without mitigating patient safety in common. However, depending on the nature of the procedure and the quality and the technical subtleties of the model, the real-life situation is simulated to a greater or lesser extent. The experience during this study convinced us that the model we had developed simulated real life conditions very well and was extremely useful for trainees without previous experience of VUS and VUS-guided endovascular procedures.

The results of this prospective randomized simulation training are limited by the small sample size and the nonblinded study design as the instructors responsible for the grading of the performance were not blinded as to whether the participants had completed their simulator training or not. The study group participants experienced additional training time and could familiarize themselves with the simulator characteristics as well as the procedural steps. This confounding factor might have positively influenced the outcomes of the study group participants, especially the less experienced trainees. The study gives reasons to assume that the developed simulation model has a construct validity as a performance difference between the experienced and inexperienced participants could be determined. However, definitive statements referring to this and its potential predictive validity need to be addressed in future studies with more participants.

The flow models used in this study produced some hyperechogenic mirror artefacts. However, by gradually adjusting the pressure to the probe and B-mode gain, the occurrence of these artefacts could be reduced to a minimum. The estimated cost of a single pAVF procedure simulation when reusing the endovascular devices wherever possible amounts to approximately EUR 100. While usage of a radiation-free imaging modality in this setting permits almost limitless training, the silicone vessels are not made for repeated use as they leak where they are punctured, which impairs the visibility of the luminal structures.

## 5. Conclusions

We developed a lifelike flow model in which the ultrasound-guided creation of a pAVF could be effectively simulated. Face and content validity of the simulation device were established, and the model was well-received by the study participants.

## Figures and Tables

**Table 1:** Independent pAVF task accomplishment.

	VUS Skills	VUS-Guided Puncture	Endovascular Device Handling	Ellipsys Catheter^®^ Handling	pAVF Dilatation
Task type	Correct identification of DCV and PRA, diameter and distance measurement	Successful puncture of PRA through DCV	VUS-guided guide-wire advancement and sheath insertion	Correct placement of Ellipsys^®^ catheter	Correct application of PTA balloon
Study group simulation training, n = 10	80%	10%	20%	0%	20%
Study group after simulation training, n = 10	90%	80%	50%	80%	80%
Control group, n = 10	90%	20%	20%	30%	50%

Notes: VUS = vascular ultrasound, pAVF = percutaneous arteriovenous fistula, DCV = deep communicating vein, PRA = proximal radial artery, PTA = percutaneous transluminal angioplasty.

## Data Availability

Not applicable.
